# Associations between self-reported lifetime history of traumatic brain injuries and current disability assessment in a population sample of Canadian adults

**DOI:** 10.1371/journal.pone.0188908

**Published:** 2018-01-05

**Authors:** Gabriela Ilie, Edward M. Adlaf, Robert E. Mann, Anca Ialomiteanu, Hayley Hamilton, Jürgen Rehm, Mark Asbridge, Michael D. Cusimano

**Affiliations:** 1 Department of Community Health and Epidemiology, Dalhousie University, Halifax, Canada; 2 Social and Epidemiological Research, Centre for Addiction and Mental Health, Toronto, Canada; 3 Dalla Lana School of Public Health, University of Toronto, Toronto, Canada; 4 Injury Prevention Research Office, St. Michael’s Hospital, Toronto, Canada; 5 Division of Neurosurgery, University of Toronto, Toronto, Canada; Universita degli Studi di Perugia, ITALY

## Abstract

**Objective:**

This study describes the association between history of lifetime traumatic brain injury (TBI) and current disabling functional restrictions among Ontario adults.

**Setting and design:**

A two-stage rolling cross-sectional sample of 6,048 adults aged 18 to 93 were interviewed by computer assisted telephone interviewing between 2011–2013 regarding their mental health and substance use in Ontario, Canada. TBI criteria were defined by loss of consciousness for minimum five minutes or at least one overnight hospitalization. Dimensions of functionality restrictions in the last 30 days were measured with the WHO Disability Assessment Schedule (WHODAS).

**Results:**

The estimated mean for global disability in this sample of Ontario adults was 2.75 (SD = 5.4, range 0–40). The estimated means of global disability for individuals who reported a history of lifetime TBI was 4.16 (SD = 7.12) and compared with 2.46 (SD = 4.98) for individuals who never had a TBI (p < 0.001). Adults with a history of lifetime TBI had greater odds of global and item disability including restricted cognition, decreased self-care, difficulties with social relationships, fewer life activities and reduced participation in society compared to adults without a history of TBI (p < 0.001), even after adjusting for values of age, sex, marital status, household income and education.

**Conclusion:**

The co-occurrence of history of lifetime TBI with self-reported disability within the past 30 days provide evidence that careful consideration, planning and understanding of short and long term health needs of TBI survivors are critical.

## Introduction

Traumatic brain injuries (TBI) represent a major cause of long term disability worldwide and are a source of global economic burden [1–7]. Main mechanisms of traumatic brain injuries include falls, sports, motor vehicle collisions, and violence [8–10]. Injuries to the head may affect brain function alone, or may occur in conjunction with spinal cord injuries leading to either temporary or permanent disability [2,11]. TBIs can affect a person’s quality of life, finances, ability to work and relationships [3,12–14]. In 2005 it was estimated that 3.32 million American civilians were living with a long-term TBI disability [1]. Military personnel, rescue workers and victims of terrorism-related attacks are also at risk of sustaining a TBI [14–16]. Some studies indicate that as many as 87% of persons in prison in the US report a history of TBIs [17–18]. Evidence indicates that history of TBI increases the incidence and severity of post-traumatic stress disorder [19]. Spielberg et al. (2015) found among a large sample of war veterans that even mild forms of TBI facilitated the re-experiencing of post-traumatic stress episodes [20]. It has been estimated that in Canada there are 41 TBI-related hospital admissions daily, mostly involving severe injury [21]. In Ontario, Canada’s second largest province, 1 in 5 adolescents ages 10 through 20, and 1 in 6 adults ages 18 through 93, report a history of TBI [10,22–23]. Survivors of moderate to severe TBI typically live for several decades post-injury with chronic life-long disabilities and, as a result, the cost in poor quality of life, family and financial burden to society is quite high [1–6,19–20;24–25]. Total economic impact of TBI in the US was estimated to be over $60 billion in 2000, with approximately $51 billion from indirect (productivity loss) costs [5]. Although the indirect cost of TBI in Canada is unknown, the Public Health Agency of Canada estimates that the total direct cost for 2000–2001 was $151.7 million [19]. History of TBI places people at higher risk of acquiring new injuries contributing to increased chance of disability and poor quality of life long term [26–31].

Developing effective care and rehabilitation strategies for TBI necessitate an understanding of how health functioning relates to TBI. While several population-based studies have identified specific health-related quality of life impediments faced by people with TBI, including those related to mental health and substance use, little evidence exists with regards to the range of disabilities that these individuals may experience [2–3,10–13,16,19–34]. The World Health Organization Disability Assessment Schedule (WHODAS version 2.0) has been developed as a global assessment of disability that can be employed internationally [35–38]. The WHODAS 2.0 is based on a comprehensive, biopsychosocial model of disability which is congruent with the International Classification of Functioning, Disability and Health (http://www.who.int/classifications/icf/en/), and has been extensively validated with somatic, psychiatric and substance use related conditions [36,39]. However, to our knowledge, to date, only two studies have used the WHODAS 2.0 to assess disability in the context of TBI [11, 26]. Kuo et al. (2015) examined health and disability using the WHODAS 2.0 among clinical samples of 1316 patients with TBI and 1348 patients with spinal cord injuries in Taiwan [11]. They found that among TBI patients there were significantly greater impediments related to poor cognition, self-care, relationships, life activities, and participation in society compared to those with spinal cord injuries. Other variables such as sex, age, income, and place of residence were also found to independently contribute to disability. Svetkova et al. (2010) assessed disability among 100 patients using WHODAS 2.0 and the International Classification of Functioning Disability and Health (ICF) and found that the most important limitations among the adults in their sample, as indicated by WHODAS, were household and work activities, as well as participation in social activities [26]. Here we report an assessment of disability, employing the short form WHODAS 2.0, experienced by a large provincial sample of Ontario adults who either reported a self-reported a history of lifetime TBI or reported never such injury in their lifetime.

## Methods

The data we used for this investigation are based on an annualized multiyear data series of 6,074 Ontario residents aged 18 or older drawn from 3 calendar-year cycles (a period spanning January 2011 through December 2013) of the *CAMH Monitor*, a rolling cross-sectional RDD telephone survey of Ontario adults, conducted for the Centre for Addiction and Mental Health (CAMH) by the Institute for Social Research (ISR), York University [40–41]. Excluded from selection were adults who were phoneless, institutionalized, and unable to complete the interview in English. Unlike most jurisdictions, in Ontario in 2010 only 0.5% of households were phoneless [41].

The resulting multiyear design employed a stratified (region × year), two-stage (telephone number followed by household member with the last birthday) probability sample drawn quarterly by the random digit dialing of listed and unlisted landline and mobile telephone numbers generated by list-assisted sampling. Each calendar year, the four quarterly non-overlapping samples are cumulated to produce a single annual dataset. Our analysis is based on 3 random subsamples totaling 6,074 respondents who were asked the TBI question of which an estimated 16.4% (95% CI: 15.2, 17.6) reported a history of lifetime TBI and 5.9% (95% CI: 3.9, 8.9) of adults reported a TBI in the past 12 months. The respective American Association for Public Opinion Research’s (AAPOR) based (eligibility-adjusted) response rate were: 51%, 51%, and 48% [41]. A metadata description of the survey and discussion of data quality and potential nonresponse bias is web available [41].

One month following the mailing of pre-notification letters to sampled members, computer assisted telephone interviews (CATI) were conducted in English at ISRs centralized and supervised CATI facility by more than 30 experienced interviewers (minimum number years of experience was 10 years), typically averaged 24–26 minutes and contained a possible 140 items. To maximize the data-to-cost ratio while minimizing the response load of a lengthy interview, the CATI employs randomized split-ballot interviews by randomly assigning sampled members to 1 of 2 concurrently administered interview panels consisting of a fixed topic set of core items and content specific to either panel. The results reported here concern exclusively panel B, the panel where our TBI items were asked (n = 6,074). Post survey assessments of the interview showed positive quality indicators. Since 2005, roughly 90% judged the interview as “not at all difficult” Even among respondents that should experience greater burden, we found that most recent immigrants and non-English home speakers similarly judged the interview as “not at all difficult” (88% and 84%, respectively).

All procedures followed were in accordance with institutional and national ethical standards on human experimentation as well as with the Helsinki Declaration of 1975, as revised in 2000. Informed consent was obtained online from all patients included in the study [40,41]. The study was approved by the Research Ethics Committees of CAMH, St. Michael’s Hospital and York University.

### Traumatic brain injury

Head injuries sustained in one’s lifetime were assessed by a single question prefaced as follows: *We are interested in any head injuries that resulted in you being unconscious (knocked out) for at least 5 minutes*, *or you had to stay in the hospital for at least one night because of it*. Respondents were then asked: *How many times*, *if ever in your life*, *have you had this type of head injury*? Responses were binary coded to represent lifetime TBI (yes = 1; no = 0). Respondents were also asked if any of these injuries were in the past 12 months (recent TBI). Responses were binary coded to indicate past 12 month TBI (yes = 1; no = 0). Similar questions assessing TBI have been used previously to assess TBI prevalence [27,30].Mechanism of injury was only assessed for TBIs which occurred in the past 12 months (last injury) due to concerns about recall for more remote TBIs. While respondents were able to participate in regular adult activities, we cannot rule out the possibility of cognitive impairments affecting responses of TBI-injured adults.

The CAMH Monitor also assessed the mechanism of injury for events that occurred in the past 12 months. Motor vehicle accident, other vehicle accident, bicycle accident, fight, sports injury, falls, other types of injury and don’t know, were the options assessed.

### Disabling functional restrictions

The abbreviated 12-item WHODAS 2.0 was used to detect disability (i.e., functional restrictions) experienced within the past 30 days [42–45]. Respondents rated the difficulty to which their restrictions interfered with their daily living in the preceding 30 days on a 5-point scale ranging from 0 (none) to 4 (extreme/cannot do). The WHODAS 2.0 consists of six domains: cognition, mobility, self-care, relationships, life activities, and participation in society [35]. As well, a global disability score was generated per WHODAS recommendations (see items and domains in Table 1). WHODAS does not employ a diagnostic cut score. Instead WHO provides two methods for scoring. In the simple method scores assigned to each of the responses: “none” (0), “mild” (1) “moderate” (2), “severe” (3) and “extreme” (4), are summed (maximum score is 48). The more complex method of scoring is called “item-response-theory” (IRT), and is calculated with a SPSS syntax (available through the WHO). This scoring is obtained converting the individual’s total score summing all 12 items, averaged against the maximum score possible (48) into a metric ranging from 0 to 100 (where 0 = no disability; 100 = full disability). According to WHO, the simple sum of the scores of the items across all domains constitutes a statistic that is sufficient to describe the degree of functional limitations [38–39]. We therefore decided to utilize the simple scoring method for reporting our results. In our sample the alpha reliability of the WHODAS was α = .87. Studies found that WHODAS’ domains and components Cronbach's α vary between 0.83 and 0.99 and Person separation index (PSI) between 0.70 and 0.95 [46–48].

**Table 1:** WHODAS 2.0 12 item simple scoring mean scores, confidence interval and standard deviations (SD) among Ontario adults, by sex and age categories, 2011–2013 *CAMH Monitor survey* (n = 5941).

	Women		Men		Total	
Age Group	*N*	*Mean (CI)*	*N*	*Mean (CI)*	*N*	*Mean (CI)*
		*SD*		*SD*		*SD*
**18–29**	254	1.79 (1.38, 2.20)	213	2.37 (1.58, 3.17)	467	2.08 (1.63, 2.54)
		.21		.41		.23
**30–39**	430	2.83 (2.30m 3.36)	303	2.07 (1.58, 2.56)	733	2.45 (2.09, 2.81)
		.27		.25		.18
**40–49**	680	2.25 (1.87, 2.62)	426	2.43 (1.86, 3.01)	1106	2.33 (2.00, 2.67)
		.19		.29		.17
**50+**	2135	3.89 (3.61, 4.18)	1500	2.71 (2.43, 2.99)	3635	3.32 (3.12, 3.52)
		.15		.14		.10

### Covariates

The following five covariates were employed: sex (male = 1); age (18–29, 30–39, 40–49 and over 50); marital status (married, partner, previously married, and never married); educational attainment (without high school completion, completed high-school, some post-secondary education completed, university degree completed); household income was based on a family’s past calendar year gross income (less than 30,000$, 30,000–49,000$, 50,000–79,000$, and 80,000$ and above). The reason for the age categorization we have selected is strictly for statistical/sample size reasons.

### Analysis

Because complex survey data typically violate the assumption of independence of observations, estimation methods that accommodate such data sampling must be used otherwise variances will be understated [48]. To accommodate the complex survey data, variances were estimated using Taylor Series Linearization available in the Complex Sample module in SPSS V20.0 [48]. The final analyses were based on a design employing normalized inclusion weights of 6048 adults drawn from18 equally allocated strata (6 regions × 3 years). Furthermore, because our TBI item was asked of a subsample, we used subpopulation estimation methods, without which estimated error would be understated. Binary logistic regression assessed the association of the 12 outcomes on TBI status (lifetime vs. not), while holding constant our five covariates. To assess the association of global disability score on TBI status, we used logistic regression without and with the five covariates. Listwise deletion reduced the global disability score estimation sample to 6,048 from 6,074. The mean age of participants was 47.2 (range: 18–93; SD = 16.51) and 48.2% were men. Variables used in our analysis held steady between 2011 and 2013.

## Results

The WHODAS 2.0 12 items distribution of mean scores by age group and sex is displayed in Table 1. Of the total population, 52.3% scored 0, that is they reported no difficulty in any activity, 27.7% of the population scored between 1 to 4, 11.2% scored between 5 to 9, and 8.8% scores between 10 to 40. The scores increased with age (Mean = 2.07, 2.45, 2.34, 3.21, for individuals aged 18–29, 30–39, 40–49, and 50+, respectively) after controlling for sex and the complexity of the design. Women (Mean = 2.99) scored slightly higher than men (Mean = 2.48) overall, and this difference remained statistically significant (Mean = 2.98 vs. 2.48, for women vs. men, respectively) after controlling for age. The mean of global disability for individuals who reported a history of lifetime TBI was 4.16 (SD = 7.12) and was statistically significantly different from the mean 2.46 (SD = 4.98) for individuals who never had a TBI. When adjusting for age, sex, marital status, education and household income, the predictive model remained statistically significant with an estimated mean of global disability for individuals with reported history of lifetime TBI of 4.10 (SD = .24; 95% CI: 3.63, 4.58) compared to 2.45 (SD = .08; 95% CI: 2.30, 2.61) for individuals without who never had a TBI.

The estimated lifetime prevalence of TBI among Ontario adults was 16.4% (95% CI: 15.2, 17.6), with 5.9% (95% CI: 3.9, 8.9) reporting an injury occurring in the past 12 months. The odds of lifetime TBI were 2.09 times greater for men than women. Men had odds 2.09 times higher (95% CI: 1.75, 2.50) than women of reporting a lifetime TBI. The most common mechanism of injury for recent TBI was the residual category “other causes” (39.9%, 95% CI: 19.6, 64.4), followed closely by falls (36.0%, 95% CI: 16.8, 61.0), sports injury (15.2%, 95% CI: 4.9,38.7), and motor vehicle collision (7.2%, 95% CI: 2.3,20.6).

**Fig 1:**
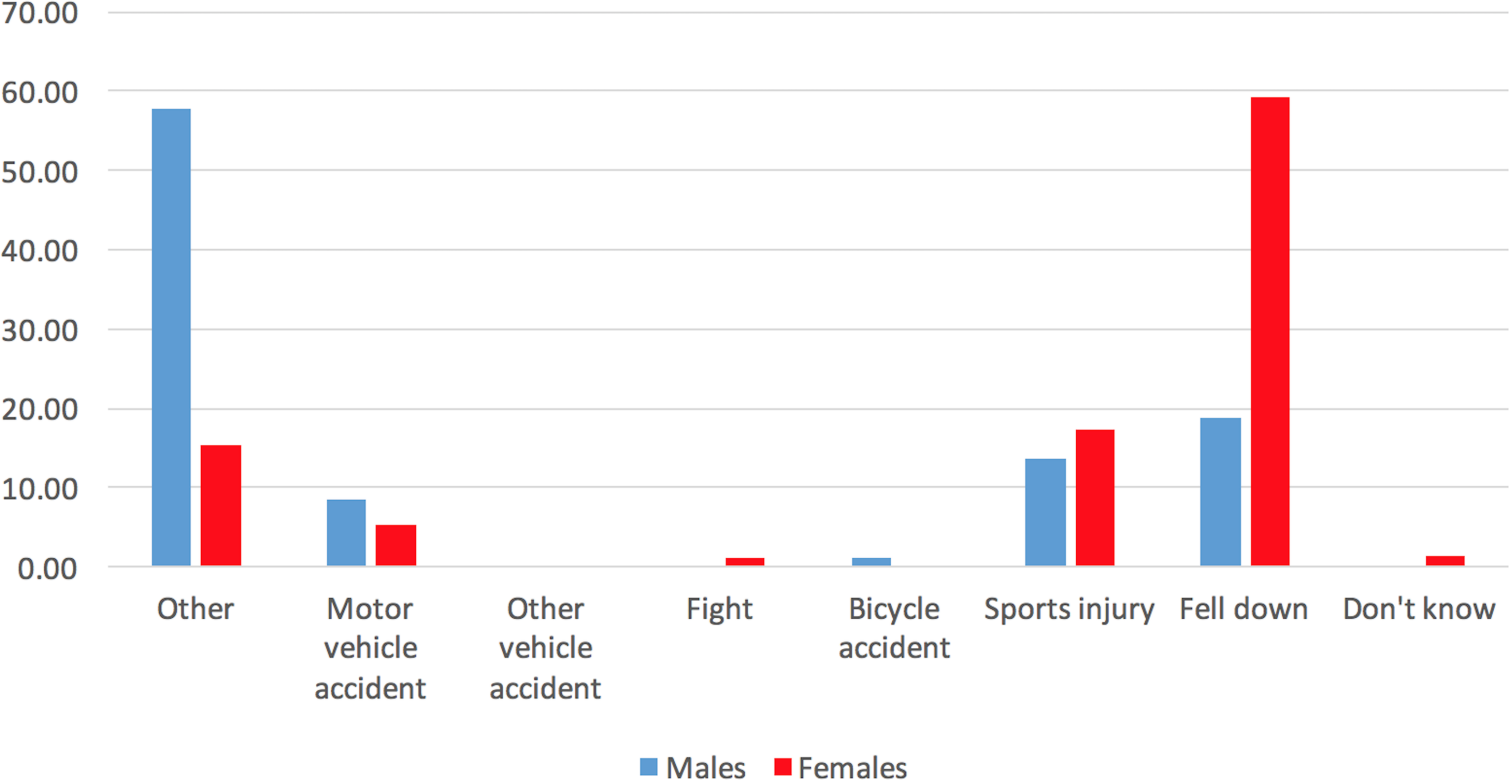
Mechanism of traumatic brain injury (TBI), by sex (in percentages) among Ontario adults, 2011–2013.

Fig 1 displays the percentages associated with each mechanism of recent TBI (incurred in the past 12 months) by sex. Main mechanisms of recent TBI for men were “other” causes followed by falls and sports injuries; for, women were falls, followed by sports injuries, and then by “other” causes. Of relevance here, we found that adults with TBI were no more likely than adults without TBI to assess questionnaire difficulty as “difficult” (10.2%; 95% CI: 6.8%, 15.0% versus 6.7%; 95% CI: 5.4%, 8.2%).

**Fig 2:**
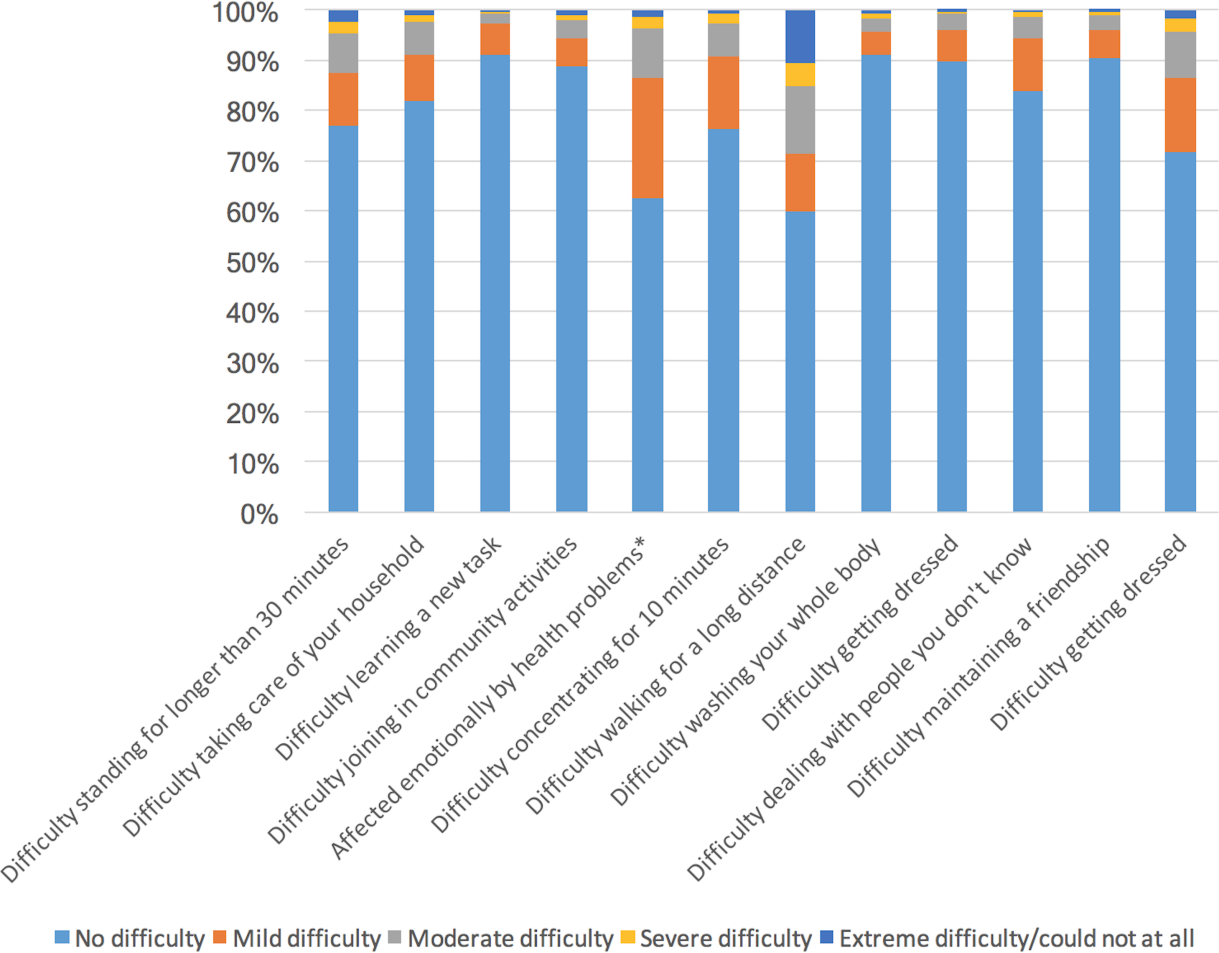
The proportion of individuals without a history of TBI who reported ‘‘no”, ‘‘mild”, ‘‘moderate”, ‘‘severe”, and ‘‘extreme” difficulty for each of disability factors experienced in the past 12 months. Note: *The wording of this item refers to the magnitude of being emotionally affected rather than amount of difficulty.

**Fig 3:**
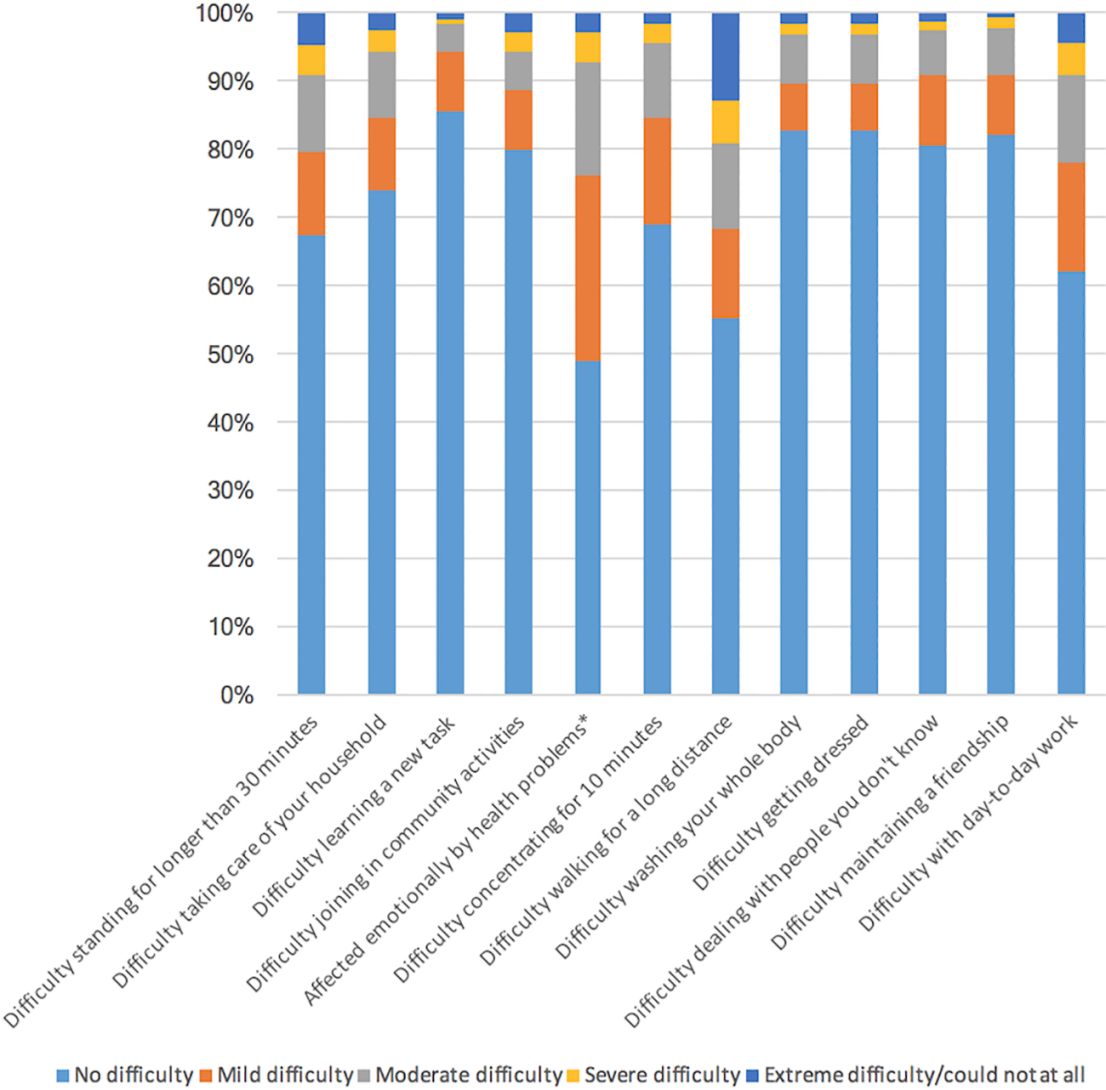
The proportion of individuals with a history of TBI who reported ‘‘no”, ‘‘mild”, ‘‘moderate”, ‘‘severe”, and ‘‘extreme” difficulty for each of disability factors experienced in the past 12 months. Note: *The wording of this item refers to the magnitude of being emotionally affected rather than amount of difficulty.

Severity by item for adults without and with a history of lifetime TBI is visualized in Figs 2 and 3, respectively. More than 45% of respondents with a history of TBI report having mild difficulty walking for a long distance and report being emotionally affected by their health condition(s). Difficulties with self-care (washing and dressing oneself), learning a new task and maintaining a relationship were reported by less than 20% of individuals with a history of TBI. Between 20% to 30% of respondents with a history of TBI reported mild difficulties taking part in community activities, taking care of their household, and dealing with people they don’t know, while between 30% to 40% of them have mild difficulties standing for longer than 30 minutes, concentrating for more than 10 minutes, and performing day-to-day work.

Table 2 shows individual logistic regression models for each of the 12 disabling restriction by TBI status (using Bonferroni adjustment for multiple comparisons). Firstly, for all 12 items history of lifetime TBI was statistically significantly associated with disability. Adults with a history of lifetime TBI reported significantly more disability during the past 30 days, compared to adults without TBI. Furthermore, compared to adults without TBI, those with previously incurred TBI showed higher adjusted odds of moderate difficulty learning a new task (AOR = 2.04), moderate to severe difficulty concentrating for 10 minutes (AOR = 2.03 and 2.64, respectively), mild to extreme difficulty standing for longer than 30 minutes (AOR = 1.35, 1.63, 2.64, and 2.40 respectively), mild to extreme difficulty walking for a long distance (AOR = 1.69, 1.61, 2.01, and 1.96, respectively), and mild, moderate or extreme difficulty washing their whole body (AOR = 1.64, 2.90 and 3.74, respectively). Adults with brain injuries also had increased adjusted-odds of mild to extreme difficulty getting dressed (AOR = 1.92, 2.39, 3.79 and 4.6,1 respectively), moderate to extreme difficulty dealing with people they don’t know (AOR = 1.87, 3.22, and 4.81, respectively), mild difficulty maintaining a friendship (AOR = 1.88), moderate to extreme difficulty with day-to-day work (AOR = 1.99, 2.06 and 4.07, respectively), moderate to extreme difficulty taking care of household chores (AOR = 1.64, 2.40, and 2.42, respectively), mild to extreme difficulty joining in community activities (AOR = 1.57, 1.33, 2.64 and 3.98, respectively) and mild to extreme difficulty affected emotionally by health problems (AOR = 1.65, 2.33, 2.78, and 2.25, respectively). Unadjusted odds did not differ substantially from the adjusted odds we reported here.

**Table 2:** Health and disability, as measured by WHODAS, among Ontario adults with (n = 981) or without (n = 5067) a history of traumatic brain injury (TBI), 2011–2013 *CAMH Monitor survey*.

	*Adults without history of TBI* *% (95% CIs)*	*Adults with history of TBI* *% (95% CIs)*	*OR (95% CI)*	*AOR (95% CI)*
*Total*	*83*.*6 (82*.*6*,*84*.*7)*	*16*.*4 (15*.*3*,*17*.*4)*		
***Cognition***				
*Difficulty learning a new task*^*a*^			*F(4*,*23687) = 3*.32^*^	*F(19*,*110157) = 5*.13^***^
No difficulty	91.3 (90.2, 92.2)	87.3 (84.6, 89.7)	1.00 (Reference)	1.00 (Reference)
Mild	6.0 (5.2–6.9)	7.4 (5.7–9.6)	1.28 (.93,1.77)	1.37 (.97, 1.90)
Moderate	2.1 (1.7,2.7)	3.9 (2.6,5.8)	1.92 (1.18,3.12)^**^	2.04 (1.24, 3.37)^**^
Severe	0.2 (0.1,0.5)	0.5 (0.2,1.5)	2.26 (.65,7.78)	2.31 (.70,7.65)
Extreme	0.3 (0.1,0.7)	0.8 (0.4,1.7)	2.56 (.85,7.75)	2.89 (.99,8.37)
*Difficulty concentrating for 10 minutes*^*a*^			*F(4*,*22550) = 7*.45^***^	*F(21*,*121407) = 4*.92^***^
No difficulty	87.8 (86.6,89.0)	81.7 (78.6,84.5)	1.00 (Reference)	1.00 (Reference)
Mild	7.4 (6.5,8.5)	8.7 (6.9,10.9)	1.26 (.95,1.69)	1.29 (.94,1.74)
Moderate	3.4 (2.8,4.2)	6.8 (5.1,9.0)	2.15 (1.49,3.10)^***^	2.03 (1.37,2.99)^***^
Severe	0.9 (0.6,1.3)	2.3 (1.3,4.1)	2.74 (1.38,5.44)^***^	2.64 (1.38,5.05)^**^
Extreme	0.4 (0.2,0.7)	0.4 (0.2,1.0)	1.15 (.45,2.93)	1.10 (.39,3.11)
***Mobility***				
*Difficulty standing for longer than 30 minutes*^*a*^			*F(4*,*23411) = 8*.39^***^	*F(21*,*118506) = 15*.20^***^
No difficulty	81.4 (80.1, 82.6)	73.5 (70.1, 76.7)	1.00 (Reference)	1.00 (Reference)
Mild	9.4 (8.5,10.4)	11.6 (9.3,14.2)	1.36 (1.04,1.78)^*^	1.35 (1.02,1.80)^*^
Moderate	6.2 (5.5,6.9)	8.5 (6.8,10.6)	1.53 (1.16,2.02)^**^	1.63 (1.22,2.17)^**^
Severe	1.5 (1.1,1.8)	3.1 (2.1,4.5)	2.35 (1.47,3.76)^***^	2.64 (1.64,4.23)^***^
Extreme	1.6 (1.3,2.0)	3.3 (2.4,4.6)	2.25 (1.49,3.39)^***^	2.40 (1.53, 3.78)^***^
*Difficulty walking for a long distance*^*a*^			*F(4*,*23676) = 7*.42^***^	*F(21*,*123762) = 15*.22^***^
No difficulty	84.6 (83.5,85.7)	76.5 (73.2,79.6)	1.00 (Reference)	1.00 (Reference)
Mild	5.3 (4.6,6.1)	7.9 (6.1,10.3)	1.66 (1.20,2.29)^**^	1.69 (1.22,2.36)^**^
Moderate	5.1 (4.5,5.8)	7.2 (5.5,9.5)	1.58 (1.14,2.18)^**^	1.61 (1.16,2.25)^**^
Severe	1.6 (1.3,2.0)	2.7 (1.8,4.2)	1.88 (1.16,3.06)^**^	2.01 (1.24,3.29)^**^
Extreme	3.4 (2.9,3.9)	5.5 (4.2,7.3)	1.81 (1.29,2.54)^***^	1.96 (1.35,2.85)^***^
***Self-care***				
*Difficulty washing your whole body*^*a*^			*F(4*,*23431) = 8*.97^***^	*F(20*,*116031) = 7*.13^***^
No difficulty	96.3 (95.6,96.8)	91.8 (89.7,93.5)	1.00 (Reference)	1.00 (Reference)
Mild	2.0 (1.6,2.5)	3.5 (2.4,5.0)	1.82 (1.16,2.87)^*^	1.64 (1.04,2.57)^*^
Moderate	1.2 (0.9,1.7)	3.4 (2.4,4.9)	2.94 (1.80,4.82)^**^	2.90 (1.72,2.87)^***^
Severe	0.4 (0.2,0.6)	0.6 (0.3,1.2)	1.76 (.70,4.40)	1.55 (.61,3.94)
Extreme	0.2 (0.1,0.3)	0.7 (0.3,1.5)	3.91 (1.53,10.01)^**^	3.74 (1.44,9.71)^**^
*Difficulty getting dressed*^*a*^			*F(4*,*23096) = 9*.51^***^	*F(19*,*109076) = 6*.59^***^
No difficulty	95.8 (95.0,96.4)	90.8 (88.6,92.6)	1.00 (Reference)	1.00 (Reference)
Mild	2.5 (2.0,3.0)	4.7 (3.5,6.3)	2.04 (1.39,2.98)^**^	1.92 (1.31,2.81)^**^
Moderate	1.5 (0.1.2.0)	3.3 (2.2,5.0)	2.40 (1.45,3.97)^**^	2.39 (1.39,4.12)^**^
Severe	0.2 (0.1,0.3)	0.5 (0.2,1.1)	3.44 (1.27,9.30)^*^	3.79 (1.27,11.33)^*^
Extreme	0.1 (0.1,0.3)	0.6 (0.3,1.6)	5.03 (1.53,16.50)^**^	4.61 (1.60,13.30)^**^
***Getting along***				
*Difficulty dealing with people you don’t know*^*a*^			*F(4*,*23157) = 5*.67^***^	*F(19*,*109076) = 6*.59^***^
No difficulty	91.7 (90.6,92.6)	86.3 (83.2,88.9)	1.00 (Reference)	1.00 (Reference)
Mild	5.2 (4.5,6.1)	6.9 (5.0,9.4)	1.40 (.96,2.02)	1.43 (.97–2.12)
Moderate	2.4 (1.8,3.1)	4.2 (3.0,6.0)	1.88 (1.19,2.97)^*^	1.87 (1.15,3.04)^*^
Severe	0.5 (0.3,0.8)	1.3 (0.7,2.4)	3.13 (1.38,7.12)^*^	3.22 (1.34,7.75)^**^
Extreme	0.3 (0.1,0.5)	1.2 (0.5,3.2)	4.99 (1.47,16.93)^*^	4.81 (1.41,16.49)^*^
*Difficulty maintaining a friendship*^*a*^			*F(4*,*23335) = 4*.67^**^	*F(21*,*121787) = 6*.57^***^
No difficulty	94.3 (93.3,95.1)	89.0 (86.0,91.4)	1.00 (Reference)	1.00 (Reference)
Mild	3.0 (2.4,3.7)	5.4 (3.8,7.7)	1.91 (1.23,2.95)^*^	1.88 (1.17,3.00)^*^
Moderate	2.1 (1.6,2.9)	3.9 (2.6,5.6)	1.91 (1.15,3.15)^*^	1.70 (1.00,2.90)
Severe	0.4 (0.2,0.7)	1.1 (0.4,2.7)	2.74 (.92,8.15)	2.50 (.80,7.82)
Extreme	0.1 (0.1,0.3)	0.6 (0.1,2.9)	4.92 (.82,29.48)	3.92 (.67,22.87)
***Life activities***				
*Difficulty with day to day work*^*a*^			*F(4*,*23214) = 10*.57^***^	*F(19*,*112351) = 4*.93^***^
No difficulty	86.6 (85.4,87.7)	78.8 (75.4,81.9)	1.00 (Reference)	1.00 (Reference)
Mild	7.7 (6.8,8.7)	9.0 (7.0,11.6)	1.29 (.94,1.77)	1.39 (1.0,1.93)
Moderate	4.0 (3.4,4.7)	7.4 (5.6,9.7)	2.02 (1.43,2.85)^***^	1.99 (1.40,2.84)^***^
Severe	1.0 (0.7,1.4)	2.1 (1.4,3.2)	2.28 (1.33,3.90)^**^	2.06 (1.14,3.75)^*^
Extreme	0.7 (0.5,1.0)	2.6 (1.5,4.5)	4.28 (2.22,8.27^)^^***^	4.07 (2.15,7.71)^***^
*Difficulty taking care of your household*^*a*^			*F(4*,*22937) = 5*.70^***^	*F(21*,*119566) = 9*.26^***^
No difficulty	83.8 (82.5,85.0)	78.3 (75.0, 81.3)	1.00 (Reference)	1.00 (Reference)
Mild	8.9 (8.0,10.0)	9.5 (7.4–12.1)	1.14 (.84,1.53)	1.23 (.91, 1.66)
Moderate	5.3 (4.7,6.1)	8.3 (6.4,10.6)	1.66 (1.22,2.27)^**^	1.64 (1.18, 2.28)^**^
Severe	1.2 (0.9,1.5)	2.4 (1.5,3.7)	2.23 (1.29,3.85)^**^	2.40 (1.39,4.12)^**^
Extreme	0.8 (0.5,1.0)	1.5 (1.0,2.3)	2.11 (1.21,3.69)^**^	2.42 (1.32,4.44)^**^
***Participation***				
*Difficulty joining in community activity*			*F(4*,*23537) = 9*.03^***^	*F(20*,*115892) = 5*.56^***^
No difficulty	89.5 (88.3, 90.5)	82.8 (79.6,85.5)	1.00 (Reference)	1.00 (Reference)
Mild	5.3 (4.6,6.1)	7.7 (6.0,9.9)	1.58 (1.15,2.18)^**^	1.57 (1.13,2.18)^*^
Moderate	3.7 (3.1,4.5)	4.7 (3.3,6.6)	1.34 (.88,2.05)	1.33 (.86,2.06)
Severe	0.8 (0.6,1.2)	2.4 (1.5,3.9)	3.06 (1.62,5.78)^**^	2.64 (1.35,5.14)^**^
Extreme	0.7 (0.5,0.9)	2.5 (1.5,4.2)	3.98 (2.11,7.54)^***^	3.98 (2.10,7.55)^***^
*Affected emotionally by health problems*^*a*^			*F(4*,*23777) = 14*.88^***^	*F(22*,*126073) = 9*.02^***^
No difficulty	66.4 (64.8,68.0)	51.7 (47.7,55.7)	1.00 (Reference)	1.00 (Reference)
Mild	22.0 (20.6,23.5)	27.3 (23.8,31.1)	1.59 (1.29,1.97)^***^	1.65 (1.32,2.06)^***^
Moderate	8.3 (7.4,9.3)	14.5 (12.0,17.4)	2.25 (1.73,2.92)^***^	2.33 (1.77,3.08)^***^
Severe	1.9 (1.5,2.4)	4.0 (2.7,6.0)	2.65 (1.63,4.31)^***^	2.78 (1.71,4.52)^***^
Extreme	1.3 (1.0,1.8)	2.4 (1.5,3.7)	2.33 (1.35,4.03)^**^	2.25 (1.25,4.055)^**^

Notes: Unadjusted odds ratios (OR) and adjusted odds ratios (AOR) were calculated using logistic regression. AOR were evaluated while holding fixed values of age, sex, marital status, family income, education

*** P < 0.001

** P < 0.01

* P < 0.05

^a^Past 30 days.

## Discussion

The results we report here indicate statistically significant greater disability among individuals with a history of TBI, than individuals who never had a TBI. These self-reports include both, hospitalized as well as non-hospitalized cases. Global disability scores in our sample faired similarly with the published literature with respect to both prevalence and characteristics [11,26,36,46,47]. History of lifetime TBI was more common among men than women, also a finding consistently reported by studies comparing individuals with history of TBI with those who never incurred a TBI [4,7–11,26,31]. Among previously injured adults, odds ratios were statistically significantly higher for all disability domains of WHODAS, compared to the control group. Indeed, covariates-adjusted odds ratios of past 30 days disability as measured by the short–form WHODAS in areas related to cognition, mobility, self-care, relationships, life activities, and participation in society were between 1.5 to 5.0 times greater for brain injured adults than those who never had a TBI. A recent study comparing adults with a history of TBI with those with spinal cord injuries also found that TBI injuries were associated with greater disability than spinal cord injuries, using the full 36-item version of the WHODAS 2.0 [11].

Although causal inferences cannot be drawn with our cross-sectional data, the associations between TBI and disability are temporally interpretable (i.e., lifetime TBI association with current/30-day disability). To our knowledge this is the first investigation of WHODAS 2.0 and TBI in a large general population sample of TBI and non-injured (TBI) adults, that captured hospitalized as well as non-hospitalized cases. This study therefore contributes to the scarce but developing literature assessing the broad impact of TBI with health and disability patient reported outcomes [11,26,42–43,46]. TBI survivors can face appreciable physical and psychological challenges [11,26,49]. There is a need to identify and predict these challenges to improve patient care and quality of life long term. Modern health care is now recognizing the importance of the perspective of the patient in informing health care short and long term [49]. Still much work remains to be done to understand the importance of the inter-relationships between health needs, patient satisfaction, and quality of life among survivors of TBI. The results we report here could therefore be informative to policymakers and their needs for future planning in this area.

These results complement studies demonstrating psychiatric and physical challenges survivors of TBI often report to have experienced as a result of their injury(s) [2–3,10,11,12–13,16,22–34]. Unfortunately, it is often the case that TBI survivors may end up in health practitioner’s offices where these challenges and issues may be identified without linking them to the history of TBI but other causes, perhaps even less related to the current assessments than the unidentified history of TBI(s) [19]. Yet, who we are as individuals and a society depends very much on the quality of our brains. Hence, these results may have relevant for health practitioners who assess disability, as they may speak to the necessity to inquire and attempt to rule out the possibility of history of TBI as a reason for the current disability identified, in those cases where it is not evident that the disability identified is TBI related [49]. Clarity and specificity in identifying the history of the patient prior to the current disability(s) identified could have relevance to the plan of care, and may require multidisciplinary approaches for these individuals, short and long term. At the same time, it is possible that our adults’ disabling restrictions may have predated the brain injury. While causal pathways cannot be established with our data it is nevertheless clinically indispensable to recognize that a patient sustaining a TBI is likely to experience coexisting disabling restrictions in various aspects of health-related quality of life, as demonstrated by our population estimates. Given that disability develops with time, it seems likely that individuals may fail to retrieve from memory TBI events that preceded their occurrence and not link the two unless prompted. As a result, physicians and emergency department staff should be attentive to the possible coexistence of these two conditions that could alter the individual’s treatment course.

The results we report here, however, are subject to various limitations. Firstly, our results are based on self-report and thus subject to recall bias (given the age range of our study participants) that may affect our prevalence estimates. Second, while our post-survey assessment of health and disability showed indicators of data quality, the survey’s response rate (51%), while considered tolerable, may nonetheless produce non-response bias [48]. Third, our study is the lack of information with regards to the temporal relationship between the report of lifetime TBI and the co-occurring disability impediments reported here. Based on our data we cannot establish whether these disabling restrictions are consequent to or subsequent to TBI. Although most clinical literature has been devoted to examining the relationship between TBI and disability post TBI, health and disability may also be linked to risk-taking behaviours that may predispose one to TBI. Fourth, our operational definition excluded milder forms of traumatic brain injury that leaves the individual confused or dazed without loss of consciousness, or resulted in loss of consciousness for less than 5 minutes. Fifth, our analyses were unable to investigate the role of onset, and relatedly, the time elapsed between the incurrence of the TBI and the onset of disability(s). Nor could we examine the possible influence of TBI-related hospitalization. Sixth, we cannot be sure the current disabilities reported are solely co-occurring with TBI, as they may be due to also other unmeasured causes (e.g., one or more diseases, other injuries, such as spinal cord injuries), which could be the case, especially for older adults. Future research should attempt to examine this possibility and model the relationship between different pathways of injury and disease that lead to current disability and assess how risk taking may potentially moderate or mediate some of these pathways. And seventh, our analysis was restricted to the occurrence of any TBI event. Our results may differ if our outcome was the number of TBI events. Future longitudinal investigations to assess the associations between such milder forms of TBI and the correlates we investigated here, as well research aimed at identifying causal pathways for these relationships is needed. As well, another stream of study would be a latent class investigation to examine levels of TBI severity and their implications for treatment and recovery.

Nonetheless, the results reported here provide important contributions, not only by building the TBI literature internationally, but also by demonstrating the nature of co-occurring TBI and disability. Adults who sustain such injuries may face enduring vocational/work impediments and challenges in maintaining relationships with one’s social network. Thus, the disabling impediments observed in our sample warrants further work to better understand and respond to the needs of adult survivors of TBI.
